# Supplementary material for: DIRseq as a method for predicting drug-interacting residues of intrinsically disordered proteins from sequences
Source: eLife. 2025 Oct 7;14:RP107470. doi: 10.7554/eLife.107470 (PMC12503486; doi:10.7554/eLife.107470)
Supplement: Supplementary file 1. [file elife-107470-supp1.docx]

**DIRseq: a method for predicting drug-interacting residues of intrinsically disordered proteins from sequences**

Matthew MacAinsh^1^, Sanbo Qin^1^, and Huan-Xiang Zhou^1,2^*

^1^Department of Chemistry and ^2^Department of Physics, University of Illinois Chicago, Chicago, IL 60607, USA

*Correspondence e-mail: [hzhou43@uic.edu](mailto:hzhou43@uic.edu)

Supplementary File 1

Supplementary File 1A. List of IDPs and drugs that bind to them

| IDP name | IDP sequence | Drug name (MW in Da) | Drug structure^a^ |
| --- | --- | --- | --- |
| p27  (UniProt P46527; residues 22-105) | EHPKPSACRNLFGPVDHEELTRDLEKHCRDMEEASQRKWNFDFQNHKPLEGKYEWQEVEKGSLPEFYYRPPRPPKGACKVPAQE | SJ403  (275.3) | 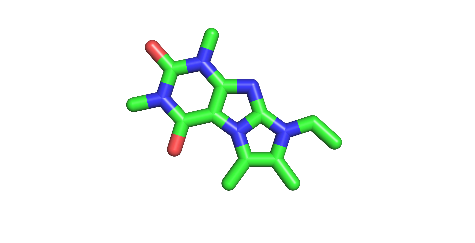 |
| p21  (UniProt P38936; residues 10-82) | QNPCGSKACRRLFGPVDSEQLSRDCDALMAGCIQEARERWNFDFVTETPLEGDFAWERVRGLGLPKLYLPTGP |  |  |
| p53  (UniProt P04637; residues 1-91) | MEEPQSDPSVEPPLSQETFSDLWKLLPENNVLSPLPSQAMDDLMLSPDDIEQWFTEDPGPDEAPRMPEAAPPVAPAPAAPTPAAPAPAPSW | EGCG  (458.4) | 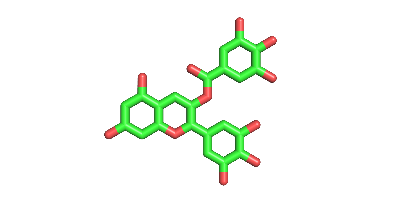 |
| α-synuclein (UniProt P37840; residues 1-140) | MDVFMKGLSKAKEGVVAAAEKTKQGVAEAAGKTKEGVLYVGSKTKEGVVHGVATVAEKTKEQVTNVGGAVVTGVTAVAQKTVEGAGSIAAATGFVKKDQLGKNEEGAPQEGILEDMPVDPDNEAYEMPSEEGYQDYEPEA | Fasudil  (291.4) | 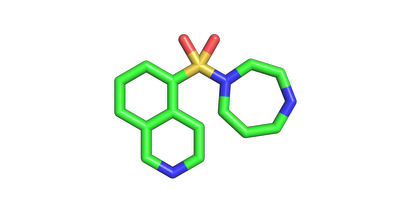 |
|  |  | Ligand-47 (323.4) | 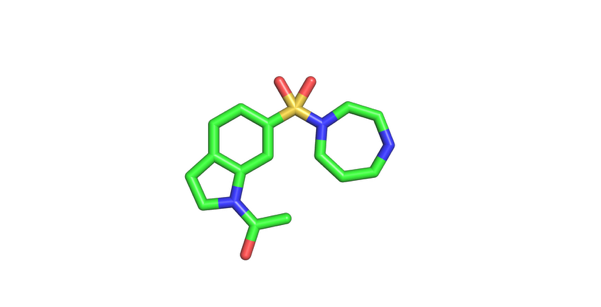 |
| Tau-5*  (BMRB 51480; residues 330-448) | AAGSSGTLELPSTLSLYKSGALDEAAAYQSRDYYNFPLALAGPPPPPPPPHPHARIKLENPLDYGSAWAAAAAQCRYGDLASLHGAGAAGPGSGSPSAAASSSWHTLFTAEEGQLYGPC | 1aa  (376.8) | 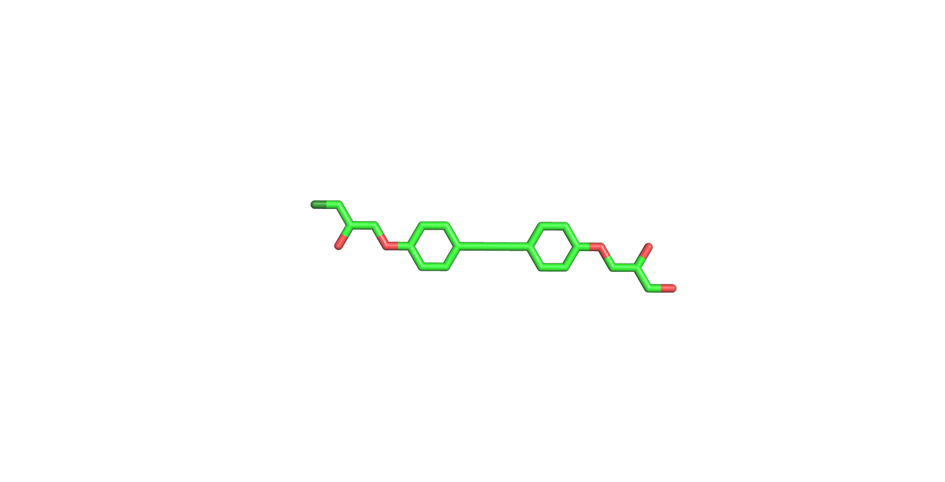 |
|  |  | EPI-001  (394.9) | 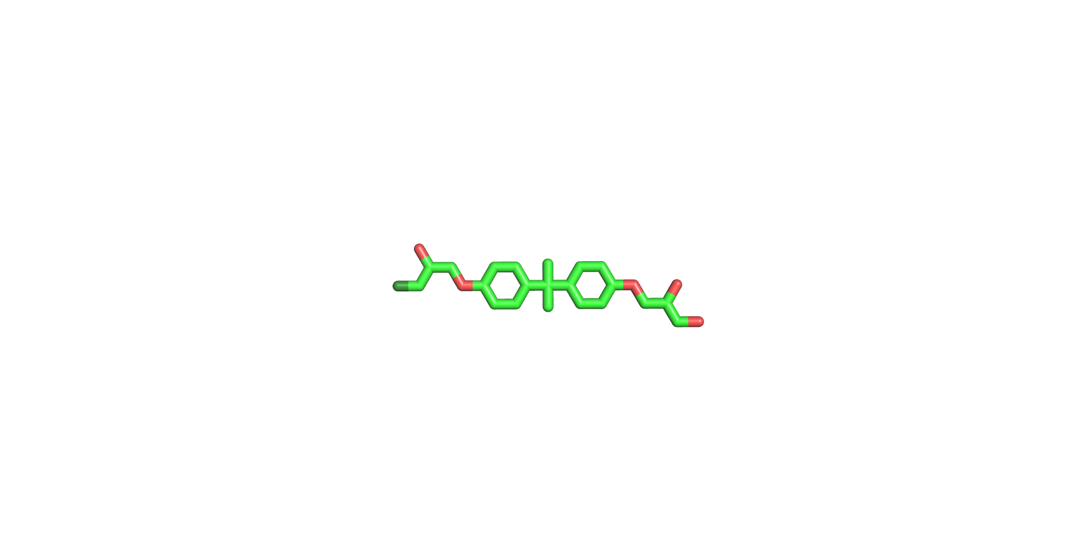 |
| NS5A-D2D3  (residues 247-466, corresponding to UniProt Q99IB8 residues 2223-2442) | SNTYDVDMVDANLLMEGGVAQTEPESRVPVLDFLEPMAEEESDLEPSIPSECMLPRSGFPRALPAWARPDYNPPLVESWRRPDYQPPTVAGCALPPPKKAPTPPPRRRRTVGLSESTISEALQQLAIKTFGQPPSSGDAGSSTGAGAAESGGPTSPGEPAPSETGSASSMPPLEGEPGDPDLESDQVELQPPPQGGGVAPGSGSGSWSTCSEEDDTTVCC | 5-Fluoroindole (135.1) | 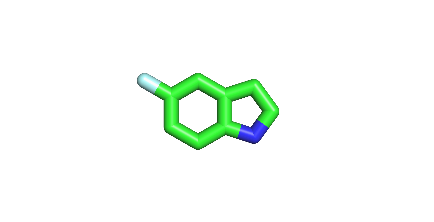 |
| β2 microglobulin  (UniParc UPI0000110347; residues 1-99) | IQRTPKIQVYSRHPAENGKSNFLNCYVSGFHPSDIEVDLLKNGERIEKVEHSDLSFSKDWSFYLLYYTEFTPTEKDEYACRVNHVTLSQPKIVKWDRDM | Rifamycin SV (697.8) | 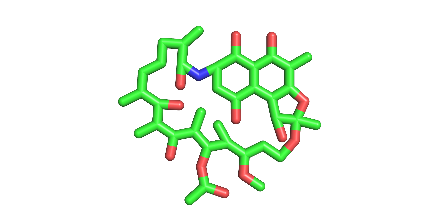 |
| hIAPP  (UniParc UPI000002B886; residues 1-37) | KCNTATCATQRLANFLVHSSNNFGAILSSTNVGSNTY | YX-I-1  (435.5) | 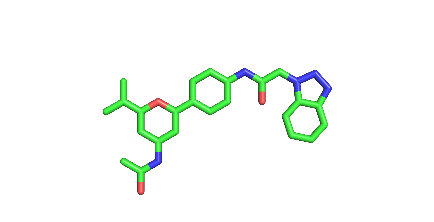 |
| Aβ42  (UniParc UPI00000315E8; residues 1-42) | DAEFRHDSGYEVHHQKLVFFAEDVGSNKGAIIGLMVGGVVIA | Myricetin (320.3) | 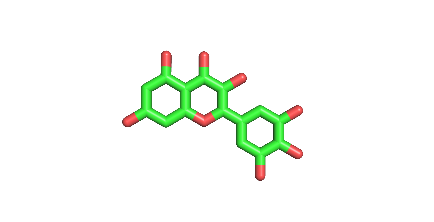 |
| c-Myc  (UniProt P01106-1; residues 363-412) | ERQRRNELKRSFFALRDQIPELENNEKAPKVVILKKATAYILSVQAEEQK | 10074-G5 (332.3) | 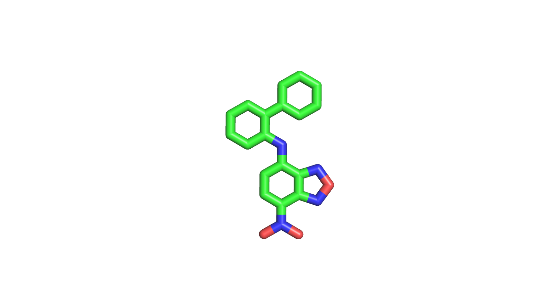 |
|  |  | 10074-A4 (409.3) | 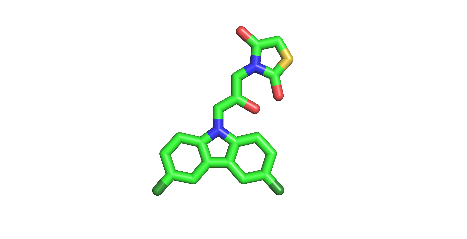 |
|  |  | 10058-F4 (249.4) | 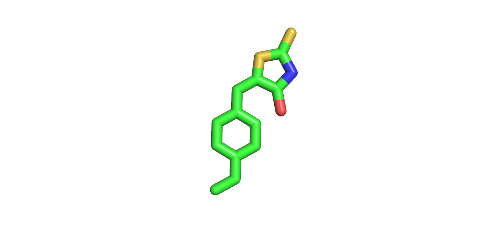 |

^a^Atom color scheme: carbon, green; nitrogen, blue; oxygen, red; sulfur, yellow, fluorine, sky blue; and chlorine, dark green.

Supplementary File 1B. Dependences of prediction accuracies on model parameters

|  | *r* | | | | *r* sum | FP | FN | TP | TP-FP |
| --- | --- | --- | --- | --- | --- | --- | --- | --- | --- |
|  | p27 | p21 | p53 | α-syn |  |  |  |  |  |
| *q* parameters | | | | | | | | | |
| seqDYN orig | 0.79 | 0.57 | 0.61 | 0.46 | 2.43 | 12 | 12 | 19 | 7 |
| DIRseq L only | 0.82 | 0.55 | 0.67 | 0.49 | 2.53 | 8 | 7 | 24 | 16 |
| DIRseq I only | 0.79 | 0.62 | 0.56 | 0.46 | 2.43 | 10 | 11 | 20 | 10 |
| DIRseq M only | 0.80 | 0.58 | 0.60 | 0.39 | 2.37 | 11 | 14 | 17 | 6 |
| DIRseq D only | 0.73 | 0.63 | 0.62 | 0.54 | 2.52 | 12 | 11 | 20 | 8 |
| DIRseq | 0.82 | 0.66 | 0.67 | 0.51 | 2.66 | 10 | 6 | 25 | 15 |
| Aromatic | 0.74 | 0.74 | 0.56 | 0.62 | 2.66 | 22 | 10 | 21 | -1 |
| CALDAVOS2 | 0.52 | 0.23 | 0.33 | 0.13 | 1.21 | 27 | 25 | 6 | -21 |
| Avg HPS scale | 0.13 | 0.01 | 0.16 | 0.14 | 0.44 | 23 | 28 | 3 | -20 |
| *b* value | | | | | | | | | |
| 0.0316 | 0.32 | 0.58 | 0.57 | 0.63 | 2.10 | 15 | 11 | 20 | 5 |
| 0.1 | 0.77 | 0.66 | 0.64 | 0.59 | 2.66 | 14 | 11 | 20 | 6 |
| 0.3 | 0.82 | 0.66 | 0.67 | 0.51 | 2.66 | 10 | 6 | 25 | 15 |
| 0.5 | 0.81 | 0.65 | 0.66 | 0.48 | 2.60 | 9 | 7 | 24 | 15 |
| 1 | 0.77 | 0.61 | 0.65 | 0.42 | 2.45 | 7 | 12 | 19 | 12 |
| 3 | 0.68 | 0.51 | 0.58 | 0.32 | 2.09 | 4 | 21 | 10 | 6 |
| *s*_1_ value | | | | | | | | | |
| 0.5 | 0.71 | 0.60 | 0.55 | 0.42 | 2.28 | 71 | 2 | 29 | -42 |
| 1 | 0.79 | 0.65 | 0.61 | 0.47 | 2.52 | 35 | 4 | 27 | -8 |
| 1.5 | 0.82 | 0.66 | 0.67 | 0.51 | 2.66 | 10 | 6 | 25 | 15 |
| 2 | 0.82 | 0.63 | 0.71 | 0.55 | 2.71 | 5 | 19 | 12 | 7 |
| 2.5 | 0.80 | 0.58 | 0.72 | 0.58 | 2.68 | 1 | 25 | 6 | 5 |
| *s*_2_ value | | | | | | | | | |
| 5 | 0.74 | 0.62 | 0.63 | 0.43 | 2.42 | 10 | 6 | 25 | 15 |
| 10 | 0.79 | 0.65 | 0.65 | 0.48 | 2.57 | 10 | 6 | 25 | 15 |
| 14 | 0.82 | 0.66 | 0.67 | 0.51 | 2.66 | 10 | 6 | 25 | 15 |
| 20 | 0.84 | 0.66 | 0.67 | 0.56 | 2.73 | 10 | 6 | 25 | 15 |
